# Designing an evidence-informed package of essential health services for Universal Health Coverage: lessons learnt and challenges to implementation in Liberia

**DOI:** 10.1136/bmjgh-2023-014904

**Published:** 2024-06-25

**Authors:** Ala Alwan, Wilhemina Jallah, Rob Baltussen, Manuel Carballo, Ernest Gonyon, Ina Gudumac, Hassan Haghparast-Bidgoli, George Jacobs, Gerard Joseph Abou Jaoude, Francis Nah Kateh, Gorbee Logan, Jolene Skordis

**Affiliations:** 1 Disease Control Priorities 3 (DCP3) Country Translation Project, London School of Hygiene & Tropical Medicine, London, UK; 2 Republic of Liberia Ministry of Health, Monrovia, Montserrado, Liberia; 3 Department for Health Evidence, Radboudumc, Nijmegen, The Netherlands; 4 International Centre for Migration, Health and Development, Geneva, Switzerland; 5 Institute for Global Health, University College London, London, UK

**Keywords:** Decision Making, Health policy, Health systems, Health services research

## Abstract

Liberia developed an evidence-informed package of health services for Universal Health Coverage (UHC) based on the Disease Control Priorities 3 evidence. This paper describes the policy decisions, methods and processes adopted for prioritisation, key features of the package and lessons learnt, with special emphasis on feasibility of implementation. Package design was led by the Ministry of Health. Prioritisation of essential services was based on evidence on disease burden, cost-effectiveness, financial risk, equity, budget impact, and feasibility of implementation. Fiscal space analysis was used to assess package affordability and options for expanding the budget envelope. The final adopted package focuses on primary healthcare and comprises a core subpackage of 78 publicly financed interventions and a complementary subpackage of 50 interventions funded through cost-sharing. The estimated per capita cost to the government is US$12.28, averting around 1.2 million DALYs. Key lessons learnt are described: (1) priority setting is essential for designing affordable packages of essential services; (2) the most realistic and affordable option when domestic resources are critically limited is to focus on basic, high-impact primary health services; (3) Liberia and many other countries will continue to rely on donor funding to expand the range of essential services until more domestic resources become available; (4) national leadership and effective engagement of key stakeholders are critical for a successful package design; (5) effective implementation is less likely unless the package cost is affordable and the health system gaps are assessed and addressed. A framework of action was employed to assess the consistency with the prerequisites for an appropriate package design. Based on the framework, Liberia developed a transparent and affordable package for UHC, but the challenges to implementation require further action by the government.

**SUMMARY BOX**

- There is a serious gap in access to essential high-impact health services at baseline. Mapping of existing services showed that only around a quarter of such services are available at the required coverage level.
- Evidence-informed prioritisation of health services can be effectively applied in low-income countries to design affordable packages of high-impact health services.
- Even when government health allocations align closely with the Abuja declaration, the limited level of government budget in some low-income countries means that the most realistic and affordable option is to focus on basic, high-impact health services at the primary healthcare level.
- Like Liberia, many low-income countries will continue to rely, at least in the short-term, on donor funding to provide free access to basic services. In these countries, considering options for mobilising more domestic resources for health should be a priority. For this purpose, full engagement of the planning and finance sectors is paramount.
- Appropriate design of Universal Health Coverage packages is critical for the transition to implementation. The framework of action recommended by the Disease Control Priorities 3 country review initiative to assess country readiness and prerequisites for package design provides a feasible approach for the successful transition to implementation.

## Introduction

Universal Health Coverage (UHC) is a global priority and a key target of the Sustainable Development Goals (SDGs).^1 2^ To achieve UHC, countries must make concerted efforts to address its three interconnected dimensions, namely: (1) improving access to a broader range of quality health services, (2) expanding coverage to the entire population and (3) reducing financial hardship.^3^ Key to advancing in these dimensions is to develop a publicly financed, essential package of high-impact health services that is free at the point of use.^4^ However, policy-makers in many low-income and lower middle-income countries (LLMICs) face major challenges in determining which services can be considered for full population access and full cost coverage within the constraints of their country’s health systems and limited fiscal space for health.

Since 2018, the Disease Control Priorities 3 (DCP3) Country Translation project, funded by the Bill & Melinda Gates Foundation, has been supporting countries through the use of evidence and model UHC packages to build technical capacity in priority setting and the design of health service packages.^5–7^ In addition to providing cost-effectiveness evidence and guidance for prioritising interventions, DCP3 also advocates for public financing of essential health services to meet the three UHC dimensions.

Recognising the importance of evidence-informed approaches in prioritising and funding essential health services, the Ministry of Health (MoH) of Liberia initiated the process of developing a UHC Essential Package of Health Services (EPHS). As a low-income country, Liberia faces intense fiscal pressures and increasing healthcare demands. In 2019, an estimated 1.98 million disability-adjusted life years (DALYs) were lost, with 61% attributed to communicable, maternal, child and nutritional conditions, 33% to non-communicable diseases (NCDs) and 5.6% to injuries.^8^ The UHC service coverage index was 45 in 2021, below the African region average of 47.^9^ Health facilities’ overall capacity to provide general health services remains suboptimal, with an average general service readiness score of 59% in 2018.^10^ The health workforce density in the public sector is currently estimated at 12.8 skilled health workers per 10 000 population.^11^ Despite increases in government health spending, a significant share of the current health expenditure is financed through out-of-pocket (OOP) payments (47%).^3^ In 2016, OOP for health pushed over 2% of the Liberian population below the extreme poverty line, and an additional 37% was pushed further into poverty.^12^

This paper presents an overview of Liberia’s experience in developing its UHC package focusing on primary healthcare (PHC). It outlines the lessons learnt and further looks at the requirements for a successful package design and feasibility of implementation.

## Package design process

The process of developing the UHC EPHS was initiated in January 2022 as part of a collaboration between the MoH and the DCP3 Country Translation Project. Package design was led by the ministry, with technical guidance and support from DCP3 experts. The process built on the experience of other countries where the DCP3 evidence had been used as a guide.^13–16^

### Timeline and key steps

A work plan was developed jointly with the MoH’s Planning Department and endorsed by the Minister of Health. The plan involved:

1. Conducting an inception workshop to build consensus, mobilise key stakeholders and agree on an operational plan and timeline.
2. Establishing a governance structure and a secretariat within the MoH, headed by the assistant minister of Health for Policy and Planning.
3. Assessing the financial mechanisms for health.
4. Mapping existing health services and selecting services for prioritisation.
5. Implementing an evidence-informed prioritisation process to identify and cost essential health services.
6. Developing implementation scenarios.
7. Reviewing the entire process and reaching consensus on the most appropriate scenario for adoption.

**Figure 1:**
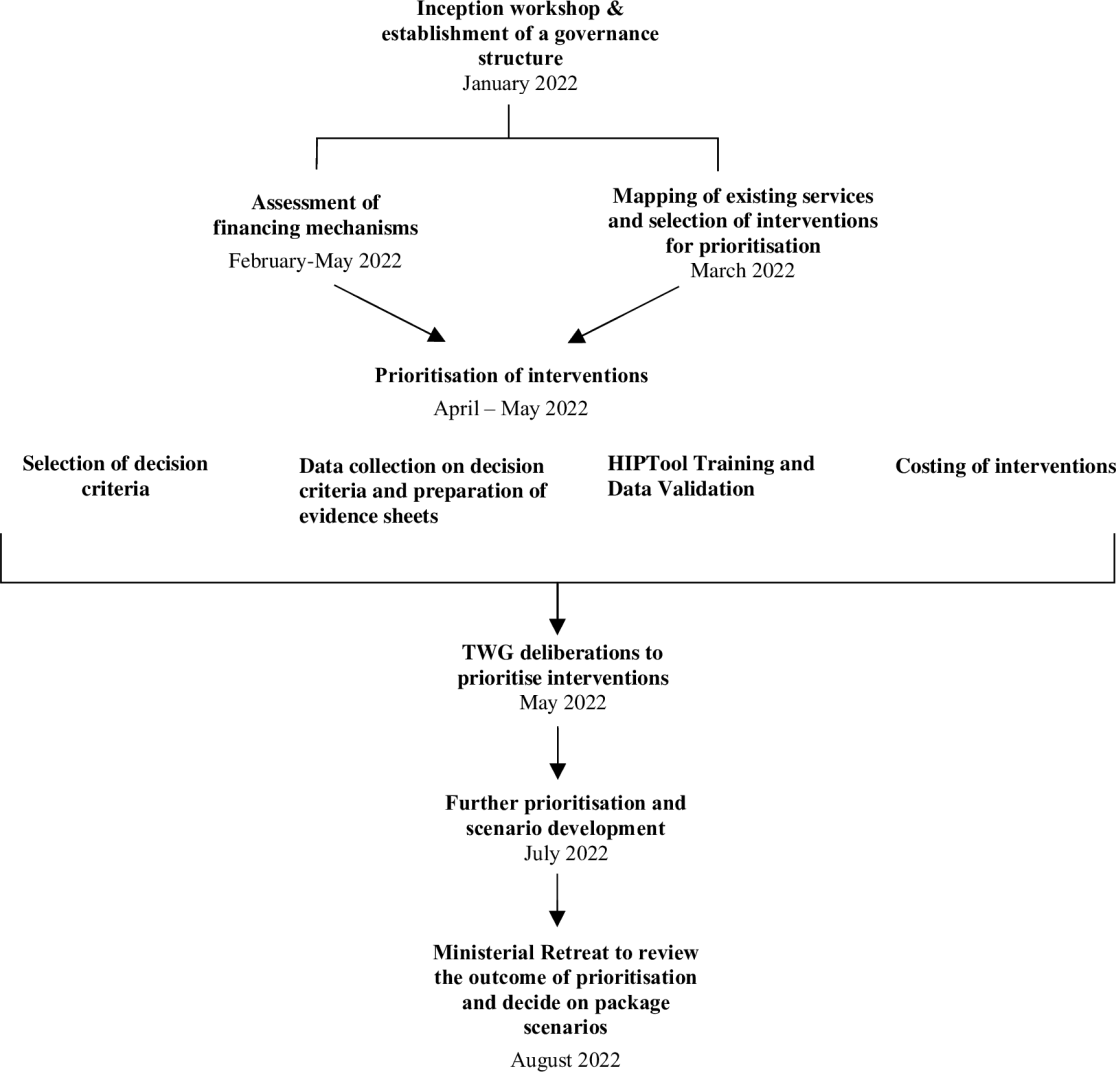
Timeline and steps followed in developing the Liberia Universal Health Coverage Essential Package of Health Services. HIPTool, Health Intervention Prioritisation Tool; TWG, technical working group.

Figure 1 shows the key steps and timeline adopted by the MoH and partners during the inception workshop.

### Establishing a governance structure

Decision-making forums were built on existing structures and included:

1. Five technical working groups (TWGs) on reproductive, maternal, newborn, child and adolescent health (RMNCAH), communicable diseases, NCDs, health system and emergency preparedness and response.
2. The health coordinating committee (HCC), chaired by the Chief Medical Officer.
3. The health sector coordinating committee (HSCC), chaired by the Minister of Health and involving key government officials and critical development partners.

**Figure 2:**
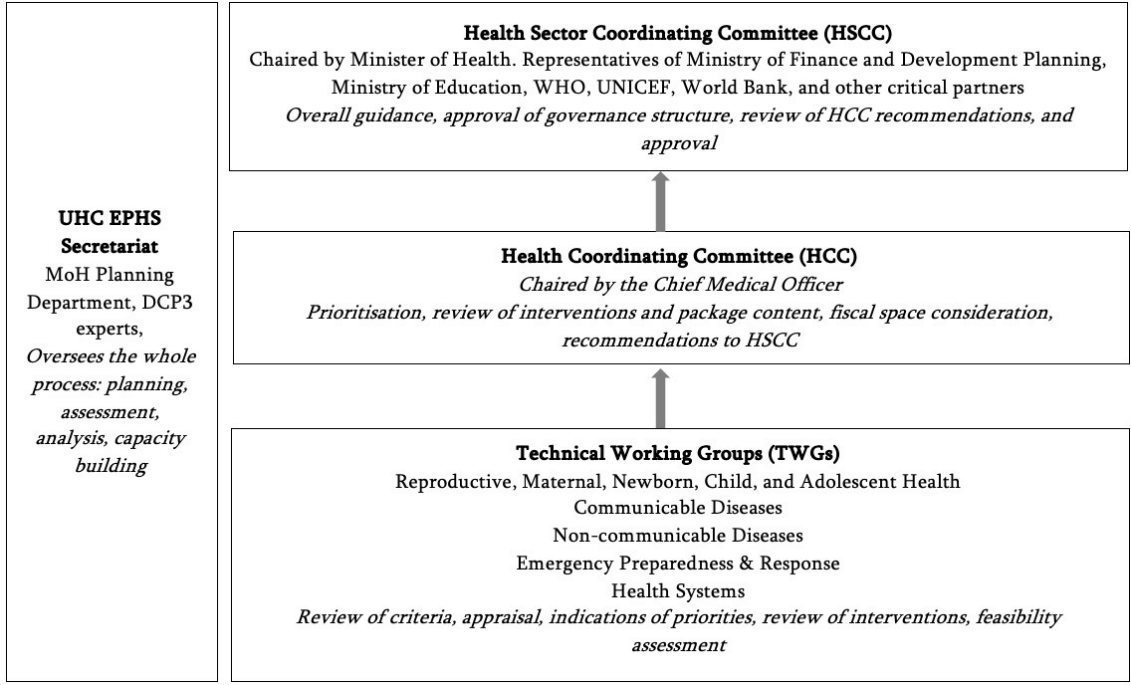
Governance structure for package design. DCP3, Disease Control Priorities 3; EPHS, Essential Package of Health Services; UHC, Universal Health Coverage.

Representatives of multilateral agencies, development partners and key non-governmental organisations (NGOs) were actively involved in all technical meetings (figure 2).

### Assessing financing mechanisms

A fiscal space analysis was conducted to assess current financing mechanisms and potential for fiscal expansion. The data used for this analysis were drawn from the International Monetary Fund 2021 World Economic Outlook Database,^17^ the MoH and the Ministry of Finance and Development Planning (MFDP). The available fiscal space estimated for the UHC EPHS was based on the total budget available from public sources, defined as the sum of government and donor budgets allocated to health.

In 2021, the government allocated 14% of its budget to health, equivalent to US$16 per capita. This is close to the Abuja Declaration’s recommendation of allocating 15% of national budgets to the health sector.^18^ However, domestic general government health expenditure accounted for only 48% of the total publicly available health budget, with the remaining 52% coming from donor funding. Further exacerbating resource constraints, challenges in public financial management (PFM) contributed to an average budget execution rate of 84%, resulting in a loss of approximately US$2–US$3 of potential per capita spending per year.

**Figure 3:**
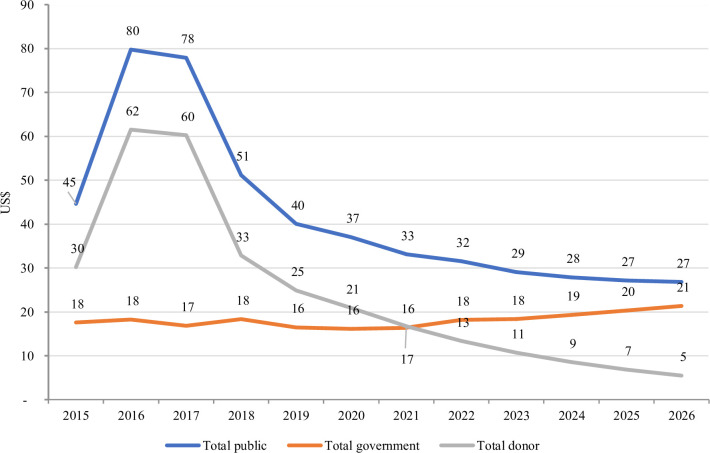
Projected per capita government and donor health funding.

In the next 5 years, the government health budget is projected to increase from US$16 per capita in 2021 to US$21 in 2026 (figure 3). However, the projected increase in government spending is insufficient to replace the expected decrease in donor funding over that period from US$17 per capita in 2021 to US$5 in 2026. Total spending on health from public sources is therefore projected to decrease from US$33 per capita in 2021 to around US$ 27 in 2026. Based on this analysis and the government’s UHC vision, the MoH planned a funding level of US$12–US$14 per capita for the publicly funded UHC package. This estimate is based on the fact that other priorities apart from package financing require funding from the government part of the fiscal space.

### Mapping existing health services and selecting services for prioritisation

Mapping of existing health services was conducted by the TWGs. The exercise aimed at addressing two questions, namely: (1) what DCP3 Essential UHC (EUHC) package interventions are relevant to Liberia’s healthcare needs and should be included in the prioritisation process? and (2) what is the estimated population coverage for the DCP3 interventions that are already provided by the health system?

Existing services were analysed against the interventions proposed by the DCP3 EUHC model package.^19^ Online supplemental table S1 shows the results of the mapping exercise. Around one-third of the 218 EUHC interventions were not provided in Liberia. Out of the remaining 151 interventions provided, it was estimated that only 25% are accessible at the required coverage level (76%–100%), while 52% were available at low coverage (1%–50%) and 23% at medium coverage (51%–75%)).

10.1136/bmjgh-2023-014904.supp1 Supplementary data

Based on the mapping exercise, the TWGs deemed 200 DCP3 interventions relevant to the Liberian context. An additional 40 services, distinct from the DCP3 interventions, were recommended by an EPHS review group in the MoH for inclusion in the prioritisation list. This addition resulted in 240 interventions shortlisted for TWG deliberations and priority setting. Many of the DCP3 interventions were adjusted to align with the country context, which resulted in modification of some interventions’ contents and/or level of care.

### Prioritising interventions

Selection of key decision criteria followed three steps. First, national policy documents were reviewed to identify key social values. Second, the DCP3 Secretariat operationalised these values in eight decision criteria, namely disease burden, effectiveness, cost-effectiveness, quality of evidence, financial risk, budget impact, feasibility, and servicing vulnerable populations. Third, a survey was conducted during a workshop with TWGs and stakeholders to define the identified criteria and rank them according to their perceived importance for Liberia.

To provide TWGs with sufficient information about the 240 shortlisted interventions, data were collated on the first six decision criteria using local, regional and global secondary sources.^20^ Information on the two remaining decision criteria—‘feasibility’ and ‘servicing vulnerable populations’—was gathered during the TWG deliberation process, described below. The Health Intervention Prioritisation Tool^21^ was used to collate and contextualise global evidence on intervention cost, cost-effectiveness, financial risk protection and estimate impact on burden of disease. This information was validated with the MoH and reported to the TWGs before a prioritisation workshop using colour-coded evidence sheets for each of the 240 interventions. An example of the evidence sheets, including definitions of decision criteria, is provided in online supplemental figure S1 and table S2.

10.1136/bmjgh-2023-014904.supp2 Supplementary data

10.1136/bmjgh-2023-014904.supp3 Supplementary data

The TWGs ranked the 240 interventions as high, medium or low priority using the framework of evidence-informed deliberative processes.^22^ This step ensured that the selection of services was objective, grounded in scientific evidence, and based on the agreed-upon decision criteria and evidence. At the initial stage, prioritisation was done without strict consideration for the available fiscal space for health. Out of 240 interventions, 132 were considered ‘high priority’. When costed, these high-priority interventions exceeded the available fiscal space for public expenditure on health. A second round of prioritisation was thus required to recommend a package that could be funded with government’s resource envelope of US$12–US$14 per capita. The 132 interventions were further refined, resulting in a final list of 128 highest-priority interventions.

### Developing package scenarios and final deliberations

Four scenarios were developed. Scenario development involved an analysis of the evidence collected for each prioritised intervention, the votes of TWGs during the prioritisation process and the extent of partner/donor funding. Funding for interventions currently receiving partial or full donor support was assumed to continue for the next 5 years. These included four fully sponsored interventions on child immunisation and prevention of mother-to-child transmission of HIV at a total cost of US$ 4.35 per capita, and 78 partially funded interventions. For partially funded interventions, the assumption was that donors contribute 30% of the cost of each partially funded intervention.

**Figure 4:**
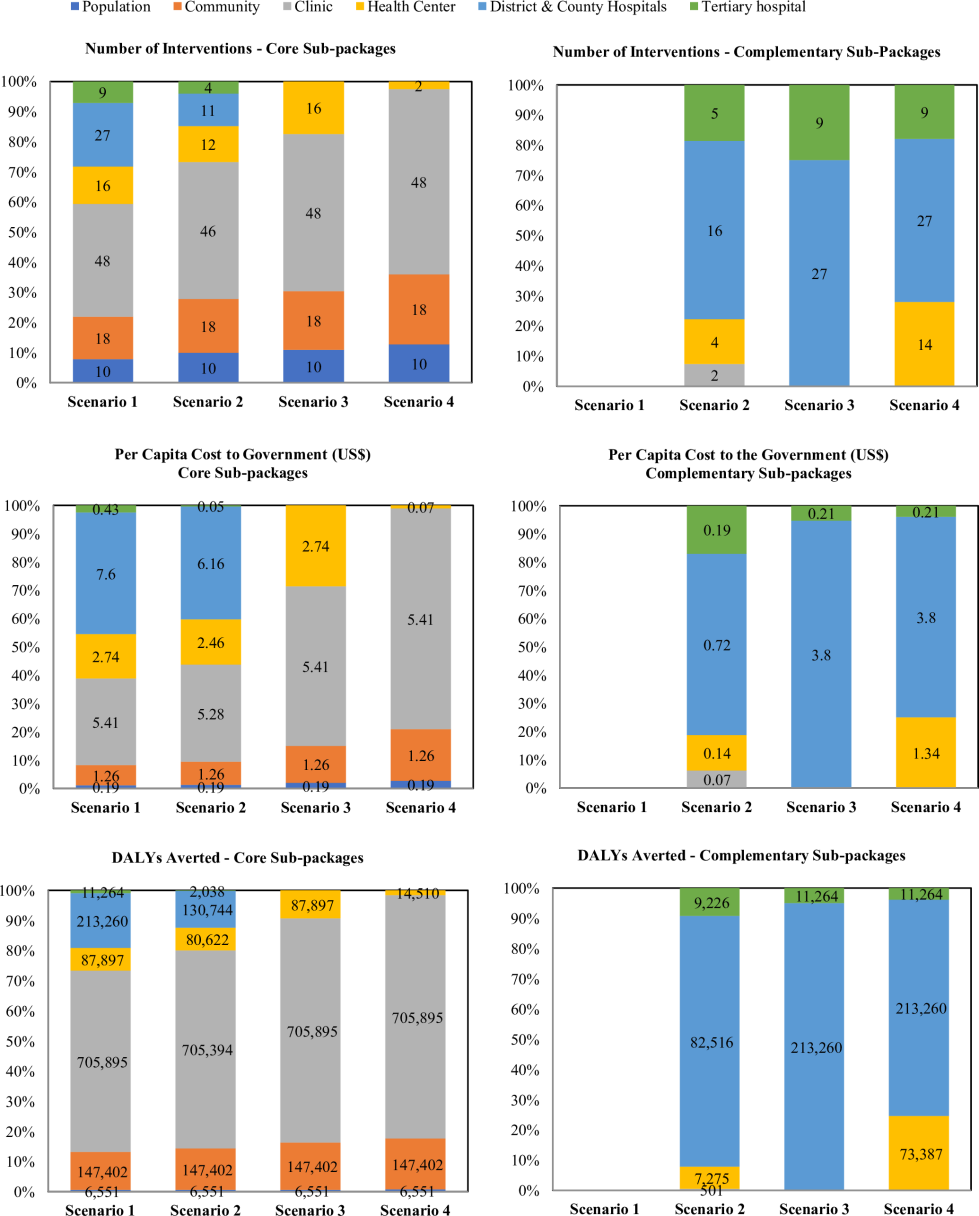
Distribution of interventions, per capita cost to the government, and DALYs averted of core and complementary subpackages in the four scenarios. DALYs, disability-adjusted life years.

Furthermore, scenario development considered the fiscal space realities, ongoing discussions with development partners in Liberia on cost-sharing options for selected interventions, and the potential introduction of a cost-sharing programme for specific service categories. A provisional cost-sharing level of 50% was initially proposed, subject to subsequent review and further consultation. The share covered by patients is currently being considered by the government during the final stages of the development of the cost-sharing programme. Figure 4 shows the distribution of interventions across the four scenarios.

Scenario 1 proposed an aspirational package, which included all high-priority interventions across six platforms, fully financed by the government. Since the cost of the package exceeds existing government spending, it could serve as a basis for advocacy and resource mobilisation or be implemented progressively as additional health resources become available.

In Scenario 2, the aim was to go through a second phase to identify the highest priority interventions that should be fully covered by government and donor funding and establish a complementary package funded through a cost-sharing programme to cover the remaining priority interventions. This scenario therefore presented a core subpackage of 101 government-funded interventions delivered through all 6 platforms and a complementary package of 27 cost-shared interventions delivered through the clinic, health centre, district and tertiary hospitals. Like Scenario 1, it also surpassed available government health spending.

Scenarios 3 and 4, in contrast, aligned with the government’s current level of health spending. Scenario 3 focused on an expanded PHC package, with core interventions provided free at point of use at population, community, clinic and health centre levels and complementary interventions provided through cost-sharing at the district, county and tertiary hospitals. Scenario 4 focused on Liberia’s definition of PHC, with core interventions provided at population, community and clinic levels. In the core subpackage, the clinic platform has the highest number of interventions, costs and DALYs averted, particularly in scenarios 3 and 4, while most of the interventions in the complementary subpackages are in the district and county hospital level and they are responsible for most of the cost.

The government’s decision on the contents of the final package was made following an in-depth analysis during a ministerial retreat organised to review the proposed scenarios. A strategic consensus was reached by the MoH leadership to use the available public funding by focusing on essential PHC services. The package included under scenario 4 was adopted during the ministerial retreat in August 2023 and subsequently endorsed by the HSCC. The selected highest-impact interventions ensure that the greatest possible health benefits are achieved within Liberia’s budget constraints.

## Final Universal Health Coverage Essential Package of Health Services

We describe here the final package, its cost to the government and potential DALYs averted. The full list of UHC EPHS interventions is provided in online supplemental table S3.

10.1136/bmjgh-2023-014904.supp4 Supplementary data

As seen in table 1, the core subpackage includes 78 interventions provided free at point of use. Almost two-thirds of the interventions will be provided at the clinic level (62%), 23% at the community level, 13% at the population level and 2% at health centres. The health centre interventions were added because they were either part of a clinic intervention requiring patient stabilisation at the health centre or were already supported by development partners to address conditions directly related to poverty. More than three-quarters of the core interventions relate to RMNCAH and communicable diseases. The core subpackage has a per capita cost to the government of US$6.93 in the first year of implementation and is expected to avert 874 359 DALYs.

**Table 1:** Breakdown of Universal Health Coverage Essential Package of Health Services interventions by platform and cluster

Platforms	Interventions, n	Total cost per capita (US$)	Cost per capita covered by partner funding (US$)	Cost-sharing (US$)	Cost per capita to government (US$)	DALYs averted
**Core**	**78**	**14.18**	**7.25**	–	**6.93**	**874 359**
Population based	10	0.27	0.08	–	0.19	6551
Community	18	1.80	0.54	–	1.26	147 402
Clinic	48	12.01	6.6	–	5.41	705 895
Health centre	2	0.10	0.03	–	0.07	14 510
**Complementary**	**50**	**13.82**	**3.12**	**5.35**	**5.35**	**297 910**
Health centre	14	3.68	1.00	1.34	1.34	73 387
District and county hospitals	27	9.70	2.1	3.80	3.80	213 260
Tertiary hospitals	9	0.44	0.01	0.21	0.21	11 264
Total	**128**	**28.00**	**10.37**	**5.35**	**12.28**	**1 172 269**

Clusters	Interventions, n	Total cost per capita (US$)	Cost per capita covered by partner funding (US$)	Cost-sharing (US$)	Cost per capita to government (US$)	DALYs averted
**Core**	**78**	**14.18**	**7.25**	–	**6.93**	**874 359**
RMNCAH	33	9.17	5.79	–	3.38	632 736
Communicable diseases	27	4.29	1.27	–	3.02	221 553
NCDs	9	0.62	0.18	–	0.44	18 094
Health system	2	0.09	–	–	0.09	1977
EPR	7	–	–	–	–	–
**Complementary**	**50**	**13.82**	**3.12**	**5.35**	**5.35**	**297 910**
RMNCAH	12	8.78	2.26	3.26	3.26	148 004
Communicable diseases	9	2.99	0.85	1.07	1.07	52 012
NCDs	11	1.05	–	0.525	0.525	23 845
Health system	18	1.00	0.01	0.495	0.495	74 049
Total	**128**	**28.00**	**10.37**	**5.35**	**12.28**	**1 172 269**

The bold represents the collective values of each of the two components of the package (core sub-package and complementary sub-package).

DALYs, disability-adjusted life years; EPR, emergency preparedness and response; NCDs, non-communicable diseases; RMNCAH, reproductive, maternal, newborn, child and adolescent health.

The complementary subpackage includes 50 interventions funded through cost-sharing. Over half of the interventions are delivered at the district and county hospitals, followed by health centre, and tertiary hospitals. The complementary subpackage has a per capita cost to the government of US$5.35. The total per capita cost to the government of the whole package (core and complementary) is US$12.28.

The final EPHS is expected to avert 1.17 million DALYs (874 000 for the core package and 297 000 DALYs for the complementary package).

## Lessons learnt and implementation challenges

The experience of Liberia in setting an evidence-informed UHC package benefitted from the outcome of the DCP3 country translation review that involved six LLMICs.^14 23–29^ The review was undertaken in 2021–2022 by a network of experts and professionals engaged in DCP3 work, who examined the experience and lessons learnt in developing or updating packages of health services to accelerate progress to UHC. The challenges encountered in these countries provided valuable guidance during the package design process in Liberia. Although some of the challenges encountered were similar to those in other countries, this section reviews important lessons learnt that are specifically relevant to the Liberia experience.

### Critical role of priority setting in reinforcing health systems and accelerating progress to Universal Health Coverage in low-income and lower middle-income countries

Despite limited previous experience in priority setting and scarcity of local evidence in Liberia, an evidence-informed prioritisation process of essential health services was effectively applied to design an affordable package of high-impact health services. By leveraging the active engagement of TWGs, the intensive training of relevant MoH staff, it is possible to conduct a systematic and stepwise process for adopting, applying and institutionalising decision criteria for prioritisation of health interventions in resource-constrained settings. However, further strengthening of national technical capacities in priority setting and related processes, including costing of interventions, are required to ensure the periodic review and updating of the UHC EPHS. Reinforcing the national health information system, including the ability to collect, generate and analyse local evidence, is important.

### Prioritisation options in countries with very limited resources for health

Although Liberia allocates 14% of its budget to the health sector, which is close to the Abuja recommendation, the available government health spending remains significantly low. As observed in other countries such as Pakistan,^13 15^ constrained fiscal resources and the government’s strategic priority to reinforce quality of services led to decisions to focus on essential PHC interventions. Even then, limited funding may prevent all PHC high priority interventions from being completely publicly financed, calling for options to mobilise additional domestic resources, particularly when donor funding is projected to decline as is the case of Liberia. In such cases, the government may be compelled to consider a complementary component of the package, with additional services provided initially through a cost-sharing arrangement until more resources are raised on the path to UHC.

### Securing sustained political commitment and leadership to Universal Health Coverage and Essential Package of Health Services

Strong high-level political commitment and effective leadership is a key prerequisite for both UHC and successful EPHS design. Liberia endorsed UHC as a target in its national health and development policies.^30–33^ Political commitment by the MoH and the resolve to develop a UHC EPHS was demonstrated by the engagement of the highest level of policy-makers in all processes and by the government’s decision to publicly finance the highest-impact PHC interventions. The Liberia experience demonstrates the need for an initial phase of political advocacy, sensitisation and partnership building before and during the inception workshop. This is necessary to convince national partners of the need for a reliable priority setting approach and an evidence-informed prioritisation of essential services. Although some parliamentarians provided support by participating in some meetings, a more formal and stronger engagement of the parliament will be necessary for sustaining political commitment and for supporting the implementation of the UHC package.

### Engaging key stakeholders

Engaging key national stakeholders and development partners is critical for national ownership of the process and for greater implementation prospects. MoH officials, representatives of UN agencies, NGOs, county health teams and development partners were engaged at all steps of the package design. However, a stronger coordination is required with other government sectors, including the planning and financing sectors. Joint work with the Ministry of Finance is essential from the outset, particularly in countries with limited public expenditure on health where considerable trade-offs are required in prioritising interventions for public financing. Although the MFDP participated in the prioritisation process and in endorsing the final package as part of the HSCC and HCC, a stronger role will be necessary in reinforcing continued package financing along the timeline of the UHC target.

The private health sector delivers a considerable part of health services in Liberia and should have been given a higher priority both during the health assessment stage and the package design process.

Civil society representatives are important stakeholders that could also have had more meaningful engagement. Such engagement would have been important in understanding how the general public perceives their key health needs and in garnering endorsement for the proposed health reforms.

### Health system assessment

Experience in other countries^14 15^ indicates that conducting a health system review is critical and that analysis of gaps and bottlenecks across the health system building blocks should be undertaken concurrently with package design to recommend health system strengthening measures and ensure a realistic package that can be feasibly rolled out. Overall, implementation is less likely if the country invests in a package development initiative without assuring adequate health system capacity.

In Liberia, the health system analysis was limited to the review of the national health vision and plan, the health financing strategy and mapping of health services. A more detailed analysis of the barriers that impede access to and optimal coverage of these services is still required. A successful transition to implementation will require a clear identification of existing gaps and a concrete and costed plan to address them.

### Requirements for proper package design and transition to sustainable implementation

Experience in many countries has identified serious gaps in the development of UHC packages, which hinder their full government endorsement and rollout. Addressing these weaknesses by meeting the requirements and prerequisites for a sound process provide better prospects for implementation and accelerated progress for UHC. The DCP3 country translation review recommended a framework of action to address the barriers that impede effective implementation. An outline of the framework is included in online supplemental table S4.

10.1136/bmjgh-2023-014904.supp5 Supplementary data

The framework has five components: securing sustained political commitment, engaging key stakeholders, assessing health systems and financing mechanisms, developing and implementing a road map and securing a successful transition to sustainable implementation. Each of the five components includes actions that need to be taken before and during the package design process.

Political commitment to UHC and the resolve to improve access to essential health services should be translated into full government endorsement of the UHC target in its national health vision and strategies. An early and serious engagement of the planning and finance sectors of government is an essential part of this commitment. Health system strengthening and adequate financing are critical challenges that often need the political backing of the parliament and joint work with the parliamentary committees on health and social services.

As described in this paper, all five components of the framework were carefully considered and followed by the Liberian MoH. Learnings from the framework were valuable in avoiding or circumventing certain weaknesses, although not all aspects have been rigorously adhered and not all actions recommended under the components have been taken. Most of the gaps in action are highlighted earlier in this section as lessons learnt or areas where government action is being taken.

As stressed before, EPHS implementation in Liberia will require continued government action to address the key gaps across the health system building blocks, particularly those related to human resources, financing, access to medicines and technologies, and health information. While the fiscal space for health was considered from the design phase, ensuring the affordability of the package beyond the initial year of implementation is the next challenge for the government. Population growth and progressive increase in interventions’ coverage over the next years of the SDG cycle will result in rising costs beyond what is currently available. Options to increase health allocations will have to be considered. One option that is currently considered by the government is to increase its low value added tax and excise tax rates coupled with efficiency gains achievable through improving PFM and strategic purchasing.

There is a strong need for institutionalisation of the process and for continued capacity building in priority setting, economic evaluation, and setting and revising EPHS.

## Conclusion

The process of defining the healthcare entitlements of the Liberian people has been at the centre of health reforms and is now serving as a major milestone for the realisation of UHC. Learning from the experience of other countries, Liberia placed special emphasis on considering from the outset the prerequisites for the transition from package design to implementation. Alignment of the package development exercise with the country’s national planning processes and building on pre-existing health system’s structures were essential enablers that facilitated the smooth implementation of the UHC package development in record time. Package design was a country-driven process, which went beyond a project-driven initiative. Decisions on priority interventions were transparent, inclusive, grounded on national values and guided by local and international evidence. The process ensured an efficient and equitable allocation of resources, targeting highest-impact interventions at the PHC level and addressing a major share of the country’s disease burden. However, stronger engagement of the finance sector and other relevant government departments will be essential for additional domestic funding and for mobilising more resources through external financing. Having endorsed the UHC EPHS, strengthening the health system to the level that allows effective package implementation is now a major challenge to the government. Engaging the private sector is one basic requirement in response to this challenge.

## Data Availability

Data are available in a public, open access repository.
