# Supplementary material for: Designing an evidence-informed package of essential health services for Universal Health Coverage: lessons learnt and challenges to implementation in Liberia
Source: BMJ Glob Health. 2024 Jun 25;9(6):e014904. doi: 10.1136/bmjgh-2023-014904 (PMC11202745; doi:10.1136/bmjgh-2023-014904)
Supplement: Supplementary data [file bmjgh-2023-014904supp001.pdf]

Supplemental Table S1: Mapping of available DCP3 EUHC interventions by platform and cluster

**Platform (1)**	**# DCP3 EUHC Interventions**	**# Available Interventions**	**1-50% Population having access**	**51 - 75% Population having access**	**76-100% Population having access**
Population-based	13	10	3 (30%)	4 (40%)	3 (30%)
Community	59	35	21 (60%)	5 (14%)	9 (26%)
Health Centre	68	52	25 (48%)	15 (29%)	12 (23%)
First Level Hospital	58	39	18 (46%)	8 (21%)	13 (33%)
Specialty & Referral Hospitals	20	15	11 (73%)	3 (20%)	1 (7%)
**Total**	**218**	**151**	**78 (52%)**	**35 (23%)**	**38 (25%)**

**Cluster**	**DCP3 EUHC Interventions**	**# Available in Liberia**	**1-50% Population having access**	**51 - 75% Population having access**	**76-100% Population having access**
RMNCAH	67	51	31 (61%)	9 (18%)	11 (22%)
CDs	52	40	15 (38%)	10 (25%)	15 (38%)
NCDs & Injuries	45	30	20 (67%)	6 (20%)	4 (13%)
Health System	54	30	12 (40%)	10 (33%)	8 (27%)
**Total**	**218**	**151**	**78 (52%)**	**35 (23%)**	**38 (25%)**

(1) Delivery platforms match definitions from the Disease Control Priorities 3 Essential UHC model package. These were later changed to fit the structure of the Liberia health system - Population-based, Community, Clinic, Health Centre, District & County Hospitals, and Tertiary Care Hospitals.
Abbreviations: RMNCAH – reproductive, maternal, newborn, and adolescent health; CDs- communicable diseases; NCDs – non-communicable diseases
