# Supplementary material for: Designing an evidence-informed package of essential health services for Universal Health Coverage: lessons learnt and challenges to implementation in Liberia
Source: BMJ Glob Health. 2024 Jun 25;9(6):e014904. doi: 10.1136/bmjgh-2023-014904 (PMC11202745; doi:10.1136/bmjgh-2023-014904)
Supplement: Supplementary data [file bmjgh-2023-014904supp002.pdf]

Supplemental Figure S1: Example of an evidence sheet

Intervention C1: Antenatal and postpartum education on family planning
Platform: Community
Cluster: Reproductive, Maternal, Newborn, and Adolescent Health

Cost-effectiveness	Burden of disease
232 $/DALY	250,922 DALYs

Targeting vulnerable groups	
Pregnant & lactating women	yes / no
Children under 5	yes / no
People with disabilities	yes / no
Prisoners	yes/ no

Feasibility	
Implementation 0-2 years	yes / no
Implementation > 2 years	yes / no

Budget impact	Effectiveness	Financial risk protection
0.008 $ per capita	163 DALYs averted	Low

Good quality of evidence
