# Supplementary material for: Designing an evidence-informed package of essential health services for Universal Health Coverage: lessons learnt and challenges to implementation in Liberia
Source: BMJ Glob Health. 2024 Jun 25;9(6):e014904. doi: 10.1136/bmjgh-2023-014904 (PMC11202745; doi:10.1136/bmjgh-2023-014904)
Supplement: Supplementary data [file bmjgh-2023-014904supp003.pdf]

Supplemental Table S2. Explanation of decision criteria

Cost-effectiveness
The value-for-money of the intervention, expressed in US$ per DALY gained.

- By choosing highly cost-effective interventions, a country maximizes its health gains for the available budget.
- A country may wish to choose some interventions that are moderately or even not cost-effective if the recommendations are strongly justified by other decision criteria like financial risk, but it should be realised that this may compromise reduce the total health gains.

Low:<br>Intervention is not cost-effective	Medium:<br>Intervention is moderately cost-effective	High:<br>Interventions is highly cost-effective

Burden of disease
The total health loss from diseases, injuries, and risk factors at the population level, expressed in DALYs lost.

- By choosing interventions targeting a high burden of disease, a country treats conditions that cause most ill-health in terms of mortality and/or morbidity.
- Note that a country may also wish to target diseases with a low burden, as these are sometimes neglected, and patients nevertheless deserve treatment

Low: intervention targeting burden of disease levels that rank in the bottom third of the prioritised interventions	Medium:<br>Intervention targeting burden of disease levels that rank in middle third of the prioritised interventions	High:<br>Interventions targeting burden of disease levels that rank in the top third of the prioritised interventions

Targeting vulnerable groups
Vulnerable groups are i) pregnant and lactating women; ii) children under the age of 5; iii) people with disabilities

- Interventions that target vulnerable groups may have special value and, for that reason, they may be considered as high-priority

TWG members to indicate whether an intervention targets a vulnerable group

Feasibility
The extent to which the intervention can be delivered through the existing health system, taking into account available human and other service resources. Note that feasibility is not about availability of funding – as these considerations are included in the criterion: costs. This criterion also includes cultural and political acceptability. Some interventions cannot be implemented immediately or within two years of the start of the implementation of the package.

Implementable > 2 yrs	Implementable 0- 2 yrs

Budget impact

The total financial implications of implementing the intervention, expressed in US$ per capita (whole Liberian population).

- By choosing interventions that are relatively cheap, more budget is available for other interventions
- Note that costs are also considered in the cost-effectiveness criterion.

High: unit cost in highest third of prioritised interventions	Medium: unit costs in middle third of prioritised interventions	Low: unit costs in bottom third of prioritised interventions

Effectiveness

The total health gains of the intervention for the population, expressed in DALYs averted.

- Interventions that produce large health gains are usually effective and target a large number of people
- Note that effectiveness is also considered in the cost-effectiveness criterion, so do not count this twice.

Low: level of health effects that rank in the bottom third of the prioritised interventions	Medium: level of health effects that rank in middle third of the prioritised interventions	High: level of health effects that rank in the in highest third of the prioritised interventions

Financial risk protection

The extent to which people can afford the cost of the intervention and are protected from catastrophic health expenditure.

- Interventions are scored on the basis of i) its costs; ii) whether it requires hospitalisation; iii) affect productivity losses.

High: Score 1-2	Medium: Score 3-4	Low: Score 5-6

Quality of evidence

The extent the evidence is relevant to the Liberia situation.

- Analysts consider quality of the study design & and relevance to setting

Poor:<br>Largely based on expert opinion	Moderate:<br>Largely based on interpretations from data from non-African region	Good:<br>Largely based on highly valid studies within the African region
