# Supplementary material for: Designing an evidence-informed package of essential health services for Universal Health Coverage: lessons learnt and challenges to implementation in Liberia
Source: BMJ Glob Health. 2024 Jun 25;9(6):e014904. doi: 10.1136/bmjgh-2023-014904 (PMC11202745; doi:10.1136/bmjgh-2023-014904)
Supplement: Supplementary data [file bmjgh-2023-014904supp004.pdf]

Supplemental Table S3: List of interventions in the Liberia's UHC Essential Package of Health Services

Code	Intervention Title	Delivery Platform	Cluster	Donor/ partner funding	Cost per capita to the government (US$)	Core or Complementary Sub-package
EPHS11	IEC/BCC on immunization	Population-Based	Communicable Disease	Partial	0.1	Core
EPHS20	IEC/BCC on preventing malaria transmission	Population-Based	Communicable Disease	Partial	0.07	Core
EPHS25	IEC/BCC on spread of filariasis, symptoms and case management	Population-Based	Communicable Disease	Partial	0.1	Core
P2	Mass media messages concerning healthy eating and exercise	Population-Based	NCD	Partial	0.01	Core
P3	Mass media messages concerning substance abuse	Population-Based	NCD	Partial	0.003	Core
P7	Conduct a comprehensive assessment of International Health Regulations (IHR) competencies using the Joint External Evaluation tool and develop, cost, finance and implement an action plan to address gaps in preparedness and response	Population-Based	EPR	No	-	Core
P8	Conduct simulation exercises and health worker training for outbreak events including outbreak investigation, contact tracing and emergency response	Population-Based	EPR	No	-	Core
P9	Decentralize stocks of antiviral medications in order to reach at-risk groups and disadvantaged populations	Population-Based	EPR	No	-	Core
P10	Develop and implement a plan to ensure surge capacity in hospital beds, stockpiles of disinfectants, equipment for supportive care, and personal protective equipment	Population-Based	EPR	No	-	Core

P11	Develop plans and legal standards for curtailing interactions between infected persons and uninfected population and implement and evaluate infection control measures in health facilities	Population-Based	EPR	No	-	Core
C4	Promotion of exclusive breastfeeding and complementary feeding by community health workers	Community	RMNCAH	Partial	0.01	Core
C8	Detection of malnourished children and referral to appropriate level of care	Community	RMNCAH	Partial	0.06	Core
C9	Detection and treatment of childhood infections (iCCM), including referral of danger signs	Community	RMNCAH	Partial	0.57	Core
C10	Education on handwashing and safe disposal of stools	Community	RMNCAH	Partial	0.01	Core
C14	Provision of vitamin A and zinc supplementation to children according to WHO guidelines	Community	RMNCAH	Partial	0.04	Core
EPHS8	Deworming of children	Community	RMNCAH	Partial	0.04	Core
C20	School based HPV vaccination for girls	Community	RMNCAH	Partial	0.11	Core
C21	Mass drug administration for lymphatic filariasis, onchocerciasis, schistosomiasis, soil-transmitted helminthiases, yaws, trachoma, and foodborne trematode infections	Community	Communicable Disease	Partial	0.46	Core
C25	Education campaigns for the prevention of gender-based violence	Community	RMNCAH	Partial	0.19	Core
C30	Provision of condoms to key populations, including sex workers, men who have sex with men, people who inject drugs, transgender populations, and prisoners	Community	Communicable Disease	Partial	0.22	Core
C32	Routine contact tracing to identify individuals exposed to TB and link them to care	Community	Communicable Disease	Partial	0.04	Core

EPHS17	DOTS and defaulters tracing	Community	Communicable Disease	Partial	-	Core
EPHS38	Mass drug distribution, administration for NTDs	Community	Communicable Disease	Partial	0.04	Core
C43	Early detection and treatment of human African trypanosomiasis and leprosy	Community	Communicable Disease	Partial	0.02	Core
C44	Total treatment for yaws	Community	Communicable Disease	Partial	0.001	Core
C45	Identify and refer patients with high risk including pregnant women, young children, and those with underlying medical conditions	Community	Communicable Disease	No	-	Core
C46	In the context of an emerging infectious outbreak, provide advice and guidance on how to recognize early symptoms and signs and when to seek medical attention	Community	EPR	No	-	Core
EPHS39	Focused use of vaccines for epidemic infections, such as COVID-19, meningococcus, Lassa fever, and others	Community	EPR	Partial	-	Core
C1	Antenatal and postpartum education on family planning	Clinic	RMNCAH	Partial	0.02	Core
EPHS3	Encouraging 8 or more ANC contacts at the facility with support from the community	Clinic	RMNCAH	Partial	0.02	Core
HC2	Management of miscarriage or incomplete abortion and post abortion care	Clinic	RMNCAH	Partial	0.0001	Core
EPHS2	Management of anaemia before and during pregnancy	Clinic	RMNCAH	Partial	0.02	Core

HC9	Screening and management of hypertensive disorders in pregnancy	Clinic	RMNCAH	Partial	0.01	Core
HC10	Screening and management of diabetes in pregnancy (gestational diabetes or pre-existing type II diabetes)	Clinic	RMNCAH	Partial	0.02	Core
FLH1	Detection and management of fetal growth restriction	Clinic	RMNCAH	Partial	0.25	Core
HC3	Management of preterm premature rupture of membranes, including administration of antibiotics	Clinic	RMNCAH	Partial	0.02	Core
HC11	Management of labour and delivery in low-risk women (BEmNOC), including initial treatment of obstetric or delivery complications prior to transfer	Clinic	RMNCAH	Partial	0.48	Core
C2	Counselling of mothers on providing thermal care for preterm newborns (delayed bath and skin-to-skin contact)	Clinic	RMNCAH	Partial	0.01	Core
C3	Management of labour and delivery in low-risk women by skilled attendants, including basic neonatal resuscitation following delivery	Clinic	RMNCAH	Partial	0.28	Core
EPHS5	Provision of vitamin A supplementation to postpartum women	Clinic	RMNCAH	Partial	0.05	Core
C6	HIV education and counselling for pregnant women, sex workers, people who inject drugs, men who have sex with men, and transgender individuals, and PLHIV and their partners	Clinic	Communicable Disease	Partial	0.01	Core
HC8	PMTCT of HIV (Option B+) and syphilis	Clinic	RMNCAH	Full	0.22	Core
C7	In high malaria transmission settings, intermittent preventive treatment in pregnancy	Clinic	Communicable Disease	Partial	0.03	Core

HC32	Provision of insecticide-treated nets to children and pregnant women attending health centres	Clinic	Communicable Disease	Partial	0.18	Core
FLH19	Management of severe malaria, including early detection and provision of rectal artesunate in community settings followed by parenteral artesunate and full course of ACT	Clinic	Communicable Disease	Partial	0.76	Core
C11	Pneumococcus vaccination	Clinic	RMNCAH	Full	1.33	Core
C12	Rotavirus vaccination	Clinic	RMNCAH	Full	0.78	Core
C13	Provision of cotrimoxazole to children born to HIV-positive mothers	Clinic	Communicable Disease	Partial	0.07	Core
EPHS6	Treatment of skin pustules or cord infection	Clinic	RMNCAH	Partial	0.0034	Core
HC1	Early detection and treatment of neonatal pneumonia with oral antibiotics	Clinic	RMNCAH	Partial	0.00018	Core
EPHS7	Treatment of neonatal tetanus	Clinic	RMNCAH	Partial	0.00003	Core
FLH3	Jaundice management with phototherapy	Clinic	RMNCAH	No	0.001	Core
C16	Childhood vaccination series (diphtheria, pertussis, tetanus, polio, BCG, measles, hepatitis B, Hib, rubella)	Clinic	RMNCAH	Full	2.03	Core
HC12	Detection and treatment of childhood infections with danger signs (IMCI)	Clinic	RMNCAH	Partial	1.07	Core
C35	In all malaria-endemic countries, diagnosis with rapid test or microscopy (including speciation) followed by treatment with ACTs (or current first-line combination)	Clinic	Communicable Disease	Partial	0.15	Core
EPHS22	Early detection and response for yellow fever	Clinic	Communicable Disease	Partial	0.0003	Core

HC30	Evaluation and management of fever in clinically stable individuals using WHO IMAI guidelines, with referral of unstable individuals to first-level hospital care	Clinic	Communicable Disease	Partial	0.93	Core
EPHS21	Diagnosis and treatment of typhoid	Clinic	Communicable Disease	No	0.02	Core
C42	Management of lymphedema	Clinic	Communicable Disease	Partial	0.79	Core
EPHS29	Case reporting and management of bites and rabies	Clinic	Communicable Disease	Partial	0.1	Core
HC16	Post gender-based violence care, including counselling, provision of emergency contraception, and rape-response referral (medical, psychosocial and judicial)	Clinic	RMNCAH	Partial	1.04	Core
HC17	Syndromic management of common sexual and reproductive tract infections (for example urethral discharge, genital ulcer, and others) according to WHO guidelines	Clinic	RMNCAH	Partial	0.4	Core
HC21	Partner notification and expedited treatment for common STIs, including HIV	Clinic	Communicable Disease	Partial	0.02	Core
HC22	PrEP for discordant couples and others at high risk of infection such as commercial sex workers (in high prevalence settings)	Clinic	Communicable Disease	Partial	0.16	Core
HC28	Screening for HIV in all individuals with a diagnosis of active TB; if HIV infection is present, start (or refer for) ARV treatment and HIV care	Clinic	Communicable Disease	Partial	0.001	Core
HC38	Provision of aspirin for all cases of suspected acute myocardial infarction	Clinic	NCD	No	0.0001	Core

HC40	Screening and management of diabetes among at-risk adults, including glycaemic control, management of blood pressure and lipids, and consistent foot care	Clinic	NCD	Partial	0.3	Core
HC42	Treatment of acute pharyngitis in children to prevent rheumatic fever	Clinic	NCD	No	0.0001	Core
HC45	Opportunistic screening for hypertension for all adults and initiation of treatment among individuals with severe hypertension and/or multiple risk factors	Clinic	NCD	Partial	0.001	Core
HC50	Management of depression and anxiety disorders with psychological and generic antidepressant therapy	Clinic	NCD	Partial	0.28	Core
HC51	Management of epilepsy, including acute stabilisation and long-term management with generic anti-epileptics	Clinic	NCD	No	0.03	Core
HC61	Resuscitation with basic life support measures	Clinic	Health System	No	0.04	Core
HC62	Suturing laceration	Clinic	Health System	No	0.05	Core
EPHS34	Early detection and treatment of eye infection	Clinic	Communicable Disease	Partial	0.02	Core
EPHS36	First aid management of eye injury	Clinic	NCD	Partial	0.001	Core
EPHS13	Management of ear infection	Clinic	Communicable Disease	No	0.02	Core
FLH12	Management of severe acute malnutrition	Health Centre	RMNCAH	Partial	0.06	Core
FLH4	Management of eclampsia with magnesium sulphate, including initial stabilization at health centre	Health Centre	RMNCAH	Partial	0.03	Core
FLH5	Management of maternal sepsis, including early detection at health centre	Health Centre	RMNCAH	Partial	0.05	Compl.

FLH7	Management of preterm labour with corticosteroids, including early detection at health centres	Health Centre	RMNCAH	Partial	0.21	Compl.
HC6	Management of neonatal sepsis, pneumonia, and meningitis using injectable and oral antibiotics	Health Centre	RMNCAH	Partial	0.004	Compl.
EPHS12	Surveillance and case reporting of immunisable diseases	Health Centre	Communicable Disease	Partial	-	Compl.
HC13	Among all individuals who are known to be HIV positive, immediate ART initiation with regular monitoring of viral load for adherence and development of resistance	Health Centre	RMNCAH	Partial	1.59	Compl.
HC26	For PLHIV and children under five who are close contacts or household members of individuals with active TB, perform symptom screening and chest radiograph; if there is no active TB, provide isoniazid preventive therapy according to current WHO guidelines	Health Centre	Communicable Disease	Partial	0.19	Compl.
HC27	Diagnosis of TB, including assessment of rifampicin resistance using rapid molecular diagnostics (UltraXpert), and initiation of first-line treatment per current WHO guidelines for drug-susceptible TB; referral for confirmation, further assessment of drug resistance, and treatment of drug-resistant TB	Health Centre	Communicable Disease	Partial	0.49	Compl.
HC33	Identify and refer to higher levels of health care patients with signs of progressive illness	Health Centre	Communicable Disease	No	-	Compl.
HC37	Low-dose inhaled corticosteroids and bronchodilators for asthma and for selected patients with COPD	Health Centre	NCD	No	0.001	Compl.
HC14	Psychological treatment for mood, anxiety, ADHD, and disruptive behaviour disorders in adolescents	Health Centre	RMNCAH	Partial	0.83	Compl.
HC52	Management of schizophrenia using generic anti-psychotic medications and psychosocial treatment	Health Centre	NCD	No	0.04	Compl.
HC57	Dental extraction	Health Centre	Health System	No	0.19	Compl.

HC60	Management of non-displaced fractures	Health Centre	Health System	No	0.05	Compl.
FLH42	Relief of urinary obstruction by catheterization or suprapubic cystostomy	Health Centre	Health System	No	0.04	Compl.
FLH9	Surgery for ectopic pregnancy	District and County Hospitals	RMNCAH	Partial	0.003	Compl.
EPHS4	Management of antepartum haemorrhage	District and County Hospitals	RMNCAH	Partial	1.49	Compl.
FLH8	Management of complications of labour, including operative delivery (CEmNOC)	District and County Hospitals	RMNCAH	Partial	0.85	Compl.
FLH6	Management of newborn complications, neonatal meningitis, and other very serious infections requiring continuous supportive care (IV fluids, oxygen, etc.)	District and County Hospitals	RMNCAH	Partial	0.01	Compl.
RH1	Full supportive care for severe preterm newborns	District and County Hospitals	RMNCAH	Partial	0.01	Compl.
FLH11	Full supportive care for severe childhood infections with danger signs	District and County Hospitals	RMNCAH	Partial	2.51	Compl.
FLH13	Early detection and treatment of early-stage cervical cancer	District and County Hospitals	RMNCAH	No	1.23	Compl.
HC47	Essential palliative care and pain control measures, including oral immediate release morphine and medicines for associated symptoms	District and County Hospitals	NCD	No	-	Compl.
HC67	Expanded palliative care and pain control measures, including prevention and relief of all physical and psychological symptoms of suffering	District and County Hospitals	Health System	No	0.004	Compl.

FLH17	Referral of cases of treatment failure for drug susceptibility testing; enrolment of those with MDR-TB for treatment per WHO guidelines (either short or long regimen)	District and County Hospitals	Communicable Disease	Partial	0.05	Compl.
FLH18	Evaluation and management of fever in clinically unstable individuals using WHO IMAI guidelines, including empiric parenteral antimicrobials and antimalarials and resuscitative measures for septic shock	District and County Hospitals	Communicable Disease	Partial	2.09	Compl.
EPHS23	Management of haemorrhagic fevers	District and County Hospitals	Communicable Disease	No	0.001	Compl.
FLH23	Medical management of acute heart failure	District and County Hospitals	NCD	No	0.05	Compl.
HC43	Long term management of ischemic heart disease, stroke, and peripheral vascular disease with aspirin, beta blockers, ACEi, and statins (as indicated) to reduce risk of further events	District and County Hospitals	NCD	No	0.71	Compl.
FLH24	Management of bowel obstruction	District and County Hospitals	NCD	No	0.004	Compl.
EPHS30	Management of head injury	District and County Hospitals	Health System	No	0.04	Compl.
FLH31	Appendectomy	District and County Hospitals	Health System	No	0.002	Compl.
FLH36	Fracture reduction and placement of external fixator and use of traction for fractures	District and County Hospitals	Health System	No	0.05	Compl.
FLH37	Hernia repair including emergency surgery	District and County Hospitals	Health System	No	0.03	Compl.
FLH39	Irrigation and debridement of open fractures	District and County Hospitals	Health System	No	0.05	Compl.

FLH44	Repair of perforations (for example, perforated peptic ulcer, typhoid ileal perforation)	District and County Hospitals	Health System	No	0.05	Compl.
FLH45	Resuscitation with advanced life support measures, including surgical airway	District and County Hospitals	Health System	No	0.02	Compl.
FLH47	Surgery for filarial hydrocele	District and County Hospitals	Health System	No	0.01	Compl.
FLH48	Trauma laparotomy	District and County Hospitals	Health System	No	0.11	Compl.
FLH49	Trauma-related amputations	District and County Hospitals	Health System	No	0.1	Compl.
FLH50	Tube thoracostomy	District and County Hospitals	Health System	No	0.03	Compl.
FLH55	Initial assessment, and prescription, and provision of individualized interventions for musculoskeletal, cardiopulmonary, neurological, speech and communication, and cognitive deficits, including training in preparation for discharge	District and County Hospitals	Health System	No	0.21	Compl.
EPHS35	Management of pneumothorax and haemothorax	Tertiary Hospital	NCD	No	0.11	Compl.
RH2	Specialized TB services, including management of MDR- and XDR-TB treatment failure and surgery for TB	Tertiary Hospital	Communicable Disease	Partial	0.01	Compl.
RH3	Management of refractory febrile illness including etiologic diagnosis at reference microbiological laboratory	Tertiary Hospital	Communicable Disease	No	0.16	Compl.

RH7	Treatment of early-stage breast cancer with appropriate multimodal approaches (including generic chemotherapy), with curative intent, for cases that are detected by clinical examination at health centre	Tertiary Hospital	NCD	Partial	0.004	Compl.
RH9	Treatment of early-stage childhood cancers (such as Burkitt and Hodgkin lymphoma, acute lymphoblastic leukaemia, retinoblastoma, and Wilms tumour) with curative intent in paediatric cancer units or hospitals	Tertiary Hospital	NCD	No	0.02	Compl.
RH11	Urgent, definitive surgical management of orthopaedic injuries (for example, by open reduction and internal fixation)	Tertiary Hospital	NCD	No	0.06	Compl.
RH13	Repair of club foot	Tertiary Hospital	NCD	No	0.04	Compl.
RH14	Cataract extraction and insertion of intraocular lens	Tertiary Hospital	Health System	Partial	0.04	Compl.
FLH57	Prevention and relief of refractory suffering and of acute pain related to surgery, serious injury, or other serious, complex, or life-limiting health problems	Tertiary Hospital	Health System	No	0.0002	Compl.

Abbreviations: Compl. – complementary sub-package; EPR – emergency preparedness and response; NCD – non-communicable disease; RMNCAH – Reproductive, maternal, newborn, child and adolescent health
