# Supplementary material for: Designing an evidence-informed package of essential health services for Universal Health Coverage: lessons learnt and challenges to implementation in Liberia
Source: BMJ Glob Health. 2024 Jun 25;9(6):e014904. doi: 10.1136/bmjgh-2023-014904 (PMC11202745; doi:10.1136/bmjgh-2023-014904)
Supplement: Supplementary data [file bmjgh-2023-014904supp005.pdf]

**Supplemental Table S4. A framework of country readiness and prerequisites for the successful design of UHC packages of essential health services**

*Securing sustained political commitment to UHC*

- Commitment and a clear government position on UHC in national health vision, strategies and plans
- Commitment at the level of the parliament
- Commitment at the sub-national level, particularly in decentralised systems
- Serious engagement of the finance and planning sectors

*Engaging key stakeholders*

- Conducting stakeholder analysis of key national players
- Building national consensus and conducting societal dialogue on health services
- Mobilising multilateral agencies and key development partners

*Assessing health system and financing mechanisms*

- Conducting an in-depth assessment of the health system, including service delivery arrangements, health workforce, and supplies, including medicines
- Mapping the health services currently provided against prioritised health services covered in model EPHS
- Assessing the fiscal space, health financing mechanisms, sustainability of health financing, and the level of public funds provided to finance the package

*Developing and implementing a road map*

- Agreeing on principles: national ownership, transparency, data-driven decision-making, and focus on feasibility and affordability
- Setting a governance structure for dialogue and deliberation
- Agreeing on decision criteria & processes for prioritising and costing interventions
- Defining the scope of the EPHS, including health delivery platforms targeted
- Prioritizing and costing interventions and linking costing to budgeting
- Establishing a monitoring framework and revising the package contents periodically

*Securing a successful transition to sustainable implementation*

- Ensuring affordable and sustainable financing along the SDG timeline
- Addressing health system gaps and reinforcing health service delivery
- Addressing the risks of instability in fragile and politically unstable contexts and instituting risk mitigation measures with stakeholders

Abbreviations: EPHS – essential package of health services; SDG – Sustainable Development Goals; UHC – Universal Health Coverage
